# Calculating Your Worth: Understanding Productivity and Value

**DOI:** 10.6004/jadpro.2014.5.2.6

**Published:** 2014-03-01

**Authors:** Todd Pickard

**Affiliations:** From The University of Texas MD Anderson Cancer Center, Houston, Texas

While health-care dollars shrink and the focus on delivering more care with less grows, it is imperative that nurse practitioners (NPs) and physician assistants (PAs) understand the key concepts of productivity and value. Furthermore, with a growing shortage of oncology physicians, the role of the advanced practitioner in solving the problem of the gap between supply and demand for oncology services has been well documented (Towle et al., 2011). Being able to clearly understand and articulate concepts of productivity and value is critical to uncovering the true contributions that NPs and PAs make to their practices. While financial contributions to the practice are important, the ability of NPs and PAs to improve patient services and access to care is equally vital.

## What Is Productivity?

Generally speaking, the concept of productivity focuses on the amount of work product created given a fixed number of resources and employees. In the health-care setting, this clearly relates to the amount of clinical services provided, the professional billing activity of the providers, and the intensity of the work. As health-care dollars continue to shrink it is to be expected that productivity of the NP and PA is an important measure. Employers in all areas of health care have to find ways to ensure that they get the maximum productivity from each and every provider. It is a matter of financial survival and sustainability.

## How Can Productivity Be Measured?

Measuring productivity in health care can be a difficult process. It is not as simple as counting the number of patients seen by each employee. Patients have a great deal of variation in their symptoms, comorbidities, and treatment options, as well as in the time and effort required to provide individual care. Additionally, there are multiple external forces that direct certain outcomes, affect minimum standards, establish safety parameters, and influence or control resource utilization. Human beings and their health needs are highly complex.

Given this complexity, there are a number of surrogates for productivity that can be utilized when analyzing health care. Simple and direct measurements such as patient volume, gross billing, or net revenue can be used. These types of measures are easy to produce and understand. However, they are significantly limited and provide an incomplete picture. These simple measures cannot account for severity of illness and acuity of care. Additionally, the professional knowledge and technical abilities required to provide care, with the associated costs of resource utilization and malpractice liabilities, are ignored. Most importantly, as will be discussed in more detail later, the NP and PA contributions to these simple measures can be hidden.

**Figure 1:**
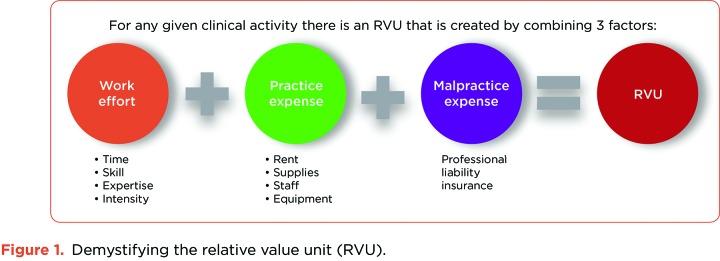
Figure 1. Demystifying the relative value unit (RVU)

In an attempt to create more accurate and sophisticated surrogates for productivity in health care, the concept of the relative value unit (RVU) was created. This measurement attempts to standardize clinician work into numerical units (Figure 1) that can be added together to create a simple measure of volume, divided per clinician in aggregate to examine productivity per provider, multiplied by conversion factors to compare work effort across surgical or medical disciplines (work RVU), and used in any number of other calculations and manipulations. The RVU was created with the intent of converting numerous factors into a single measurable unit. These factors include the time it takes to perform a given service, the technical skill that service requires, the mental effort and judgment required of the providers, and the liability risk associated with that service.

The cornerstone of the RVU is the assignment of a Current Procedural Terminology (CPT) code to every clinical service that is provided. The CPT code is used to describe medical, surgical, and diagnostic services. These codes communicate uniform information about services and procedures among providers, coders, patients, accreditation organizations, and payers for administrative, financial, and analytical purposes. Perhaps more importantly, CPT codes are used by the Centers for Medicare and Medicaid Services to determine reimbursement.

To illustrate these concepts, let us look at a simple clinical scenario. A patient presents to a PA for a routine follow-up for an established diagnosis of hypertension. The patient has no new problems, symptoms, or physical exam findings. This scenario corresponds to CPT code 99213: an office visit for an established patient. The RVU for this CPT code is 0.97. The practice can now use this information to compile productivity for the PA in question and compare this to all of the other providers in the practice.

## Benefits and Pitfalls of Productivity Measures

One of the clear benefits of productivity analysis that is based on CPT codes and RVU is that it is standardized. The work is given the same amount of credit no matter who provides the care. Therefore, physicians, NPs, and PAs are all accounted for identically when a CPT code and RVU is used to measure them. Even if reimbursement rates may be discounted for NPs and PAs, the CPT and RVU are not. It is always the same unless it is manipulated by some other factor.

**Figure 2:**
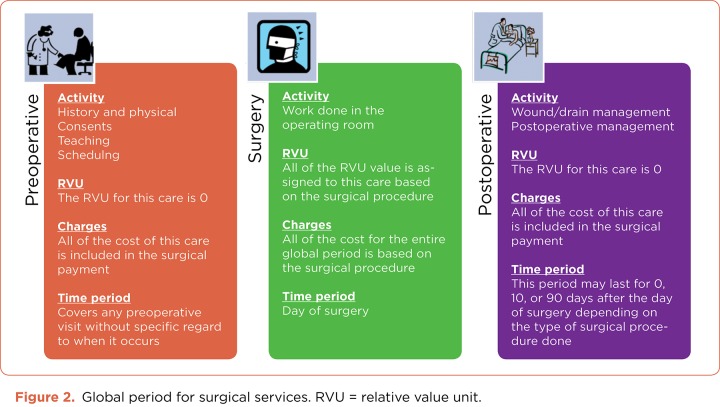
Figure 2. Global period for surgical services. RVU = relative value unit.

While this level of standardization can be beneficial, there are some nuances to consider that can impact productivity using the CPT and RVU system. For NPs and PAs in surgical practice, it is important to understand the concept of global visits (Figure 2). When a patient is treated surgically, a certain amount of the pre- and postsurgical care is considered to be included in the surgical fees (Marriott, 2010). Therefore, global visits associated with the surgery are assigned an RVU of 0. It would be a gross mischaracterization to say that the NP’s or PA’s productivity is 0 if they spend all or even a majority of their time providing pre- and postsurgical care to patients. Unless the surgical fee is apportioned to take into account the contribution of the NP or PA, it can give a much-skewed result.

The CPT and RVU system can also hide the productivity of the NP or PA. For example, the concepts of "shared visits" and "incident to" are utilized to allow practices to code and bill clinical services under the physician regardless of who actually provided the care (Marriott, 2011). For instance, if an advanced practitioner was seeing the same hypertension patient in the preceding example as "incident to" the established diagnosis and without new symptoms or physical examination findings, the practice can maximize the reimbursement for this clinical work by coding and billing it under the physician. In this scenario all of the productivity of the NP is hidden. Additionally, some payers do not enroll NPs and PAs in their plans and allow practices to code and bill under the physician. While this practice is not based upon any national policy, it is very common to have work done by the NP or PA billed under the physician when the NP or PA is not enrolled in the plan. This practice varies by state and by payer (American Association of Physician Assistants, 2011a). Both "incident to" and billing under the physician are legitimate and supported by a significant number of payers’ reimbursement policies. Yet both scenarios hide the productivity of the NP and PA unless specific internal accounting practices are utilized to ensure that NP and PA productivity is captured. Shared visits and "incident to" visits can be difficult to track and have accurate value assigned.

There are numerous other pitfalls associated with using the CPT and RVU systems without any other considerations. When clinical services are directed by scheduling or assignment, the NP and PA have no capacity to work harder. They simply see the patients who are assigned to them. If they are carrying a lighter load due to this practice, their productivity will reflect it. In capitated systems, patient panel size may be more relevant than CPTs and RVUs. If the NP and the PA are unaware of the health-care plans utilized, it is exceedingly difficult for them to be aware of their productivity and what factors they can change to positively impact this productivity. Additionally, information is only as good as the system used to collect and analyze it. If productivity is being captured by filling out paper charge slips, coders using outdated guidelines, or practice administrators using manual calculations, there are many more opportunities for productivity measures to be inaccurate or incomplete due to human error.

## Why Do Productivity Measurements Matter?

Generally speaking, productivity measurements matter because in order to understand utilization of clinical services and staff work efforts to be able to identify when change is necessary, there have to be some baseline metrics to describe what is actually happening and who is doing it. It is simply impossible to improve something that has not been quantified or qualified. At a very basic level productivity measurements can (1) provide a way to compare clinicians to their peers, (2) provide information to determine if the NP’s or PA’s work is a "cost center" or "revenue center," (3) help identify when additional clinical staff is needed, (4) be used to make determinations of compensation and bonus structures, and (5) promote transparency, accountability, and efficient management when used properly (Dean & Gans, 2012).

## How Is Value Different From Productivity?

When productivity is considered, the concepts of volume, cost, work effort, and revenue are the key factors that influence the final result. Value, on the other hand, is something quite different. It can be measured by the perceived or actual benefits gained despite costs. The concepts of quality, efficiency, effectiveness, and patient satisfaction are the key factors that influence the final result. For instance, a physician who is capable of providing a large volume of clinical encounters per day for highly complicated patients may be able to generate a very large number of RVUs. However, if the physician is abrupt and does not listen to patient concerns, poor patient satisfaction can result. In that case the physician brings very little value to the patients and eventually the practice itself.

There are a number of ways to define and create measurements of value. Examples of these methods include using instruments that measure patient satisfaction, such as Press Ganey surveys; meeting practice guidelines or performance metrics related to cancer care as published by the Association for Healthcare Research & Quality, the American Society of Clinical Oncology, and the National Comprehensive Cancer Network; reviewing patient adherence with treatment recommendations; and reviewing provider outcomes for morbidity and mortality. What is important to realize is that although NPs and PAs provide many benefits to practices that do not result in increased measures of productivity, these activities are crucial to providing care and value for both patients and the practice.

## What Brings Value?

There are numerous clinical activities that bring value to a practice (Table 1). The NP and PA can provide services that are typically provided by physicians. Ogunfiditimi, Takis, Paige, Wyman, and Marlow (2013) found that up to 30% of the work completed by NPs and PAs do not generate RVUs in a time and motion study completed at an academic medical center. Just the fact that a practice has an NP or a PA on staff can increase access to the practice for patients. Patients will have increased appointments available to them; this creates greater patient satisfaction and thus greater value. In surgical oncology practices, the physicians can focus their efforts on time in the operating room knowing that the NP or the PA will be in the office providing those pre- and postoperative services that are imperative to good patient care but do not generate RVUs. Physician assistants can also assist in surgical cases and bill for those services, allowing the physician to complete more complicated and time-consuming operations. NPs and PAs are also exceptionally situated to coordinating care in an increasingly complex health-care system. Oncology practices spend a large amount of time and effort coordinating with referring physicians, infusion treatment centers, and hospitals as well as performing peer-to-peer insurance reviews. With their added clinical expertise and ability to give direction to staff and write orders, the NP or PA can free up physicians and provide services that other staff members cannot provide. All of these functions bring unique value to the practice, the physicians, and the patients.

**Table 1:**
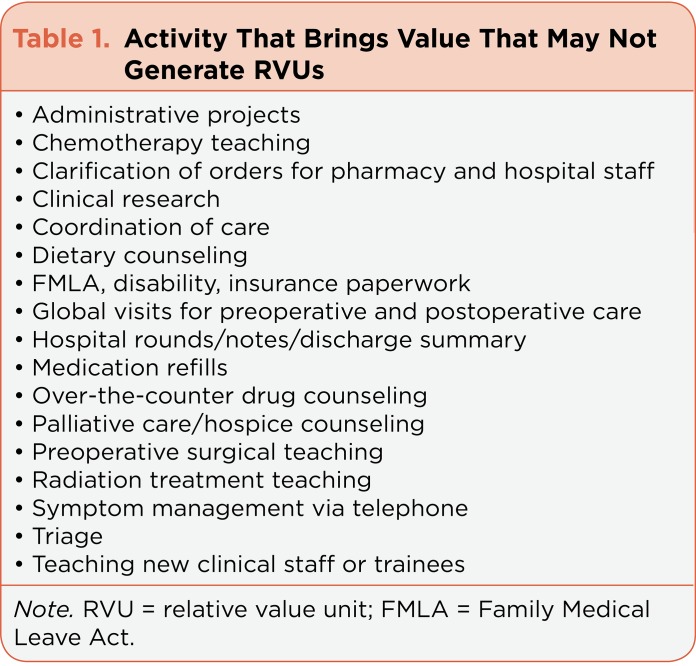
Table 1. Activity That Brings Value That May Not Generate RVUs

The NP and the PA are also adept at providing value by improving patient wait time for appointments, ensuring continuity of care, monitoring treatments to ensure adherence, empowering patients to manage symptoms through education, and prescribing medications. While these activities are obviously important to the patient, what is not as obvious is the value that this brings to the practice and the physicians. Quality of life is improved for all of the staff members in the practice. Nurse practitioners and PAs are recognized and fully authorized providers of medical care. They can provide opportunities for physicians to take time off, share call, and increase hospital coverage. Productivity is just the tip of the iceberg. Looking deeper at the entire picture illuminates the tremendous value generated by the NP and the PA.

## What Should NPs and PAs Do?

Knowing what productivity and value are is the first step for the NP and PA to be able to understand what they can and do bring their practice, but that is not enough (Table 2). It is crucial to understand all of the elements in the language of productivity (see Table 3). Just as NPs and PAs had to learn medical terminology to become effective providers, they must now learn the language of billing, reimbursement, and insurance; take an active part in the business side of the practice; talk to the coders and the office manager, and ask them for feedback on how clinical documentation can be improved to support coding that truly takes into account the complexity and acuity of patients; and take an interest in more than just the clinical practice.

**Table 2:**
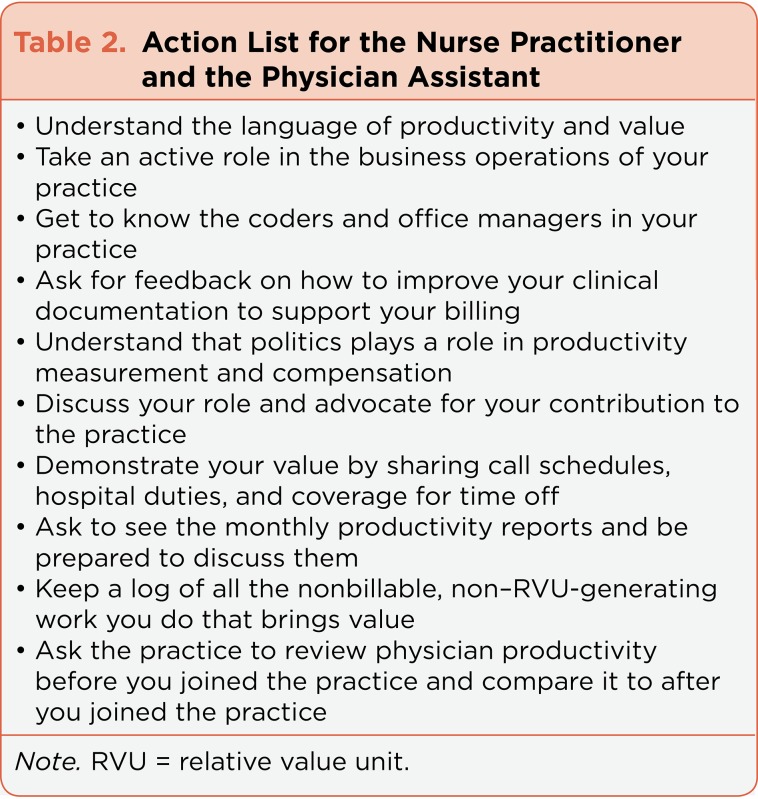
Table 2. Action List for the Nurse Practitioner and the Physician Assistant

**Table 3:**
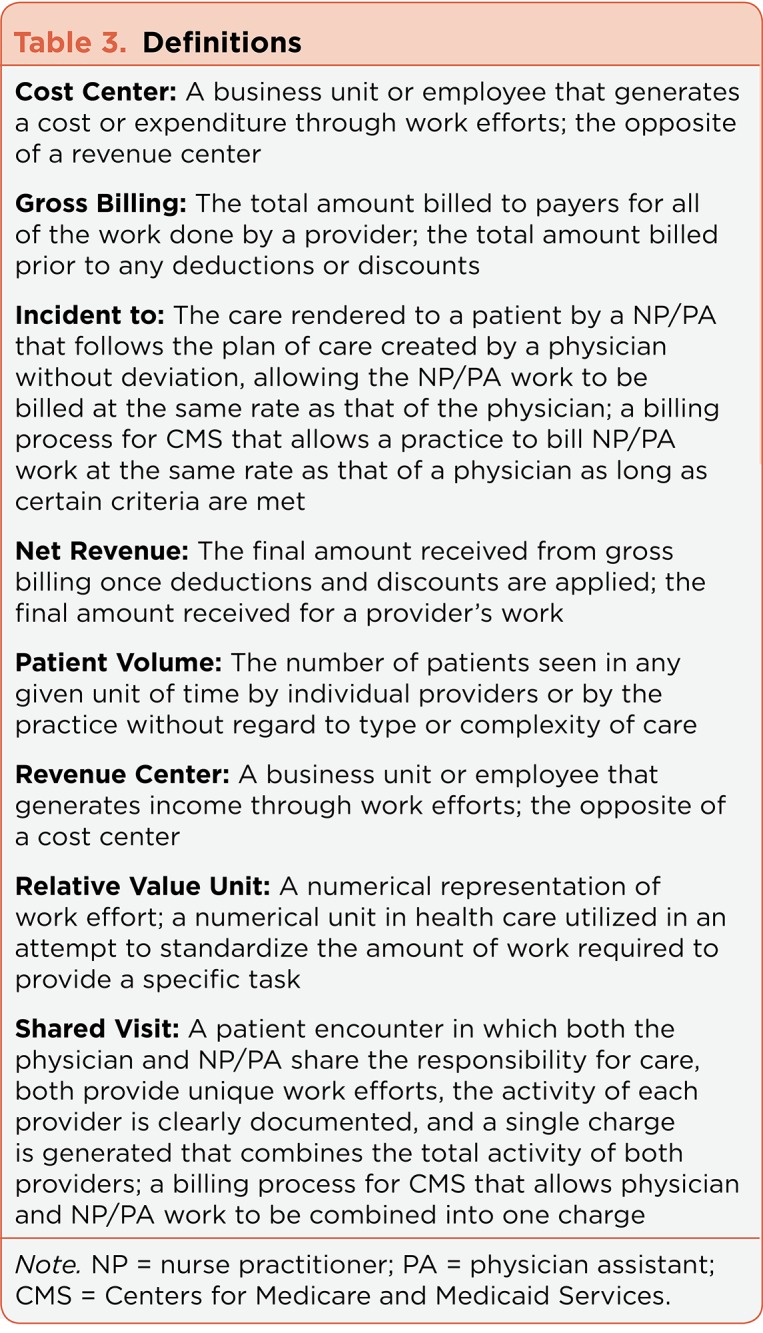
Table 3. Definitions

It is important to recognize that there are some sensitive issues related to productivity. Physicians have historically been the revenue centers for their practices. There are some growing pains and political considerations to take into account now that NPs and PAs have entered into the productivity equation. While it is clear that NPs and PAs can increase productivity, it is their value that can ease these sensitive issues. If the NP and PA are seen as partners on the team and not competitors for productivity, all sides benefit. Although this can be complicated and may require some finesse, communication and planning will go a long way toward easing any physician concerns. Discussing clinical operations, call schedules, time off, sharing of hospital duties, and other items can demonstrate NP and PA value to the physicians in the practice.

One of the most effective habits an NP or a PA can develop is being an active participant and staying involved in the productivity discussion. One way to begin this process is to ask to see the monthly productivity reports and to ensure that the practice is aware of all clinical and nonclinical activity that brings value. It can be useful to keep a log of all of the nonbillable work completed. One suggestion might be for the practice to examine physician productivity before and after the NP or PA was added to the practice. After a reasonable orientation and training period, it is likely that physician productivity will increase as the NP and the PA provide services that free up the physician’s time to focus on highly productive clinical activity.

## Conclusion

Now, more than ever, NPs and PAs need to be educated about what they bring to the table. Value to physicians, patients, and practices is more than simple measures of productivity. It is clear that advanced practitioners can provide clinical services that generate RVUs, but it takes a deeper look to understand the true value that they bring. It is important to remember that state laws or institutional policies that limit the ability of NPs and PAs to work at the top of their license can negatively impact productivity. Organizational culture, physician attitude, and utilization of good practice models are also factors that can enhance or inhibit NP and PA productivity. There is little doubt that NPs and PAs generate revenue. However, it takes active participation, ongoing monitoring, and advocacy on the part of the NP and PA to ensure that their true value is understood and appreciated. 
